# PI3K pathway mutations are associated with longer time to local progression after radioembolization of colorectal liver metastases

**DOI:** 10.18632/oncotarget.15278

**Published:** 2017-02-11

**Authors:** Etay Ziv, Michael Bergen, Hooman Yarmohammadi, F. Ed Boas, E. Nadia Petre, Constantinos T. Sofocleous, Rona Yaeger, David B. Solit, Stephen B. Solomon, Joseph P. Erinjeri

**Affiliations:** ^1^ Interventional Radiology Service, Department of Radiology, Memorial Sloan Kettering Cancer Center, New York, USA; ^2^ Department of Radiology, Mount Sinai Hospital, New York, USA; ^3^ Gastrointestinal Oncology Service, Department of Medicine, Memorial Sloan Kettering Cancer Center, New York, USA; ^4^ Human Oncology and Pathogenesis Program, Memorial Sloan-Kettering Cancer Center, New York, USA; ^5^ Marie-Josee and Henry R. Kravis Center for Molecular Oncology, Memorial Sloan-Kettering Cancer Center, New York, USA; ^6^ Genitourinary Oncology Service, Department of Medicine, Memorial Sloan-Kettering Cancer Center, New York, USA

**Keywords:** biomarkers, colorectal, PI3K, radioembolization, MAPK

## Abstract

**Purpose:**

To establish the relationship between common mutations in the MAPK and PI3K signaling pathways and local progression after radioembolization.

**Materials and Methods:**

Retrospective review of a HIPAA-compliant institutional review-board approved database identified 40 patients with chemo-refractory colorectal liver metastases treated with radioembolization who underwent tumor genotyping for hotspot mutations in 6 key genes in the MAPK/PI3K pathways (KRAS, NRAS, BRAF, MEK1, PIK3CA, and AKT1). Mutation status as well as clinical, tumor, and treatment variables were recorded. These factors were evaluated in relation to time to local progression (TTLP), which was calculated from time of radioembolization to first radiographic evidence of local progression. Predictors of outcome were identified using a proportional hazards model for both univariate and multivariate analysis with death as a competing risk.

**Results:**

Sixteen patients (40%) had no mutations in either pathway, eighteen patients (45%) had mutations in the MAPK pathway, ten patients (25%) had mutations in the PI3K pathway and four patients (10%) had mutations in both pathways. The cumulative incidence of progression at 6 and 12 months was 33% and 55% for the PI3K mutated group compared with 76% and 92% in the PI3K wild type group. Mutation in the PI3K pathway was a significant predictor of longer TTLP in both univariate (p=0.031, sHR 0.31, 95% CI: 0.11-0.90) and multivariate (p=0.015, sHR=0.27, 95% CI: 0.096-0.77) analysis. MAPK pathway alterations were not associated with TTLP.

**Conclusions:**

PI3K pathway mutation predicts longer time to local progression after radioembolization of colorectal liver metastases.

## INTRODUCTION

Patients with metastatic colorectal cancer (mCRC) have poor prognosis with 5-year survival rates between 10-20%[1]. Improvements in survival over the past ten years may be in part due to the introduction of molecular therapies including monoclonal antibodies targeted against receptor tyrosine kinases (RTK) such as the epidermal growth factor receptor (EGFR)[2]. Despite these improvements, patients with mutations in downstream effectors of the EGFR signaling pathway may not respond to these anti-EGFR antibodies [3].

**Figure 1:**
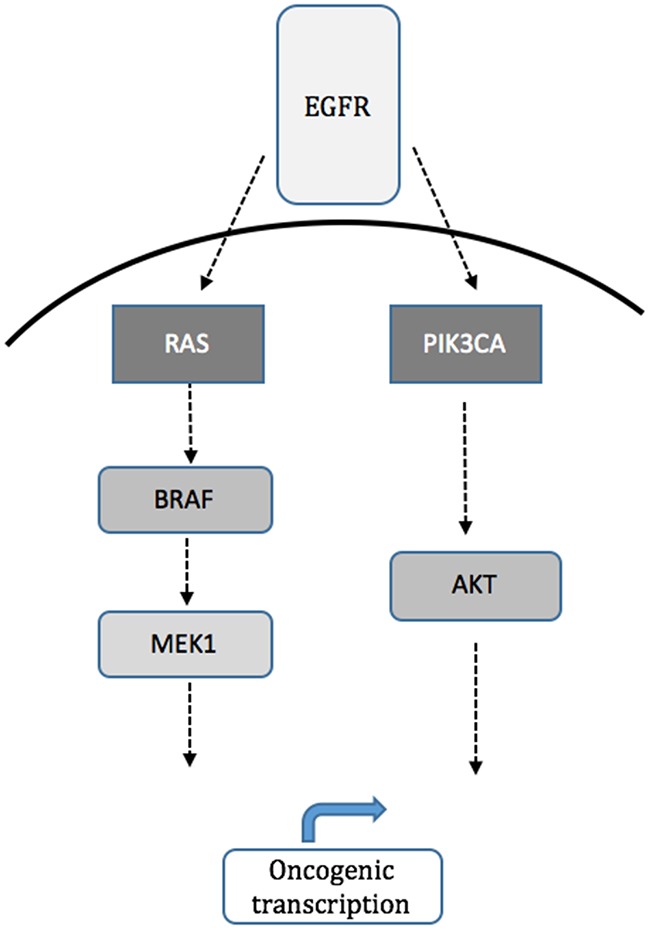
Schematic of MAPK (RAS/RAF/MEK) and PI3K (PIK3CA, AKT1) signaling pathways downstream of EGFR Although pathways run in parallel there is cross-talk and feedback as well.

Two well-established downstream effectors of EGFR are the mitogen-activated protein kinase (MAPK) and phosphatidylinositol 3-kinase (PI3K) signaling pathways (see Figure 1). Mutations in these signaling pathways are commonly seen in CRC and include activating mutations in *KRAS*, *NRAS*, *BRAF* and *MEK1* in the MAPK pathway, and in *PIK3CA*, which includes the catalytic subunit of PI3K, and *AKT1*, the key downstream effector of PI3K, in the PI3K pathway. Reported frequencies of mutations in these genes in mCRC patients are approximately 40% for *KRAS*, 20% for *PIK3CA*, 5% for BRAF, 2% for *NRAS*, and 1% for *AKT1* [4–6]. The value of MAPK mutations in predicting clinical benefit from anti-EGFR antibodies in mCRC is now well-established [7], making mutation testing part of current routine clinical care. Mutations of the MAPK and PI3K signaling pathways may also predict worse outcome independent of anti-EGFR antibody treatment [8, 9].

Y90 radioembolization (RE) is widely used as a salvage therapy for unresectable, chemorefractory colorectal liver metastases (CLM)[10–12]. A multicenter phase II clinical trial found that RE produced an objective response or disease stabilization in patients with advanced unresectable and chemorefractory mCRC and demonstrated a significant survival benefit for responders vs non-responders [13]. Response to RE in chemorefractory mCRC is quite variable and difficult to predict [14–16]. In a recent study, investigators demonstrated *KRAS* mutation status as an independent poor prognostic factor for overall survival after RE [17]. But patients with KRAS mutant mCRC have increased lung, brain, and bone metastases [18, 19], so it is unclear if this result is just a reflection of more aggressive and advanced disease in these patients.

Radiation resistance and sensitivity in relation to the MAPK and PI3K signaling pathways have been investigated in the radiation oncology literature. For example, tumors with *KRAS* mutations may be less likely to demonstrate pathologic complete response to chemoradiation [20]. Selective inhibition of the PI3K signaling pathway has been shown to increase radio-sensitivity of human carcinoma cell lines [21]. The effect of mutations in the MAPK and PI3K signaling pathway genes on tumor response to RE remains unknown. The purpose of this retrospective study was to evaluate the effect of MAPK and PI3K pathway mutation status on tumor response to salvage RE in patients with heavily pretreated CLM.

## RESULTS

Patient and tumor characteristics are summarized in Table 1. There were 40 patients with median age 60 years (range 33-82), 23 men and 17 women, 28 ECOG=0 and 12 ECOG=1 or 2. There were 19 patients who had undergone prior hepatic resection, and 24 patients who had received hepatic arterial infusion pump therapy. All patients had been treated with first line chemotherapy and 36 patients had been treated with second line chemotherapy. 20 patients received a VEGF inhibitor (bevacizumab) and 16 patients received an EGFR inhibitor (cetuximab or panitumumab). The mean tumor volume was 281 cm^3^ (range, 6-1790 cm^3^), the mean size of the largest lesion was 4.9 cm (range, 1.6-15 cm) and the mean pretreatment CEA was 1032 (range, 3-23938). There were 22 patients with >3 tumors and 18 patients with ≤ 3 tumors. There were 9 (22.5%) patients who reached stasis during radioembolization and for which dose delivery was not completed (accounting for total technical success of 77.5%).

**Table 1:** Patient, treatment, and tumor characteristics

Patient Characteristic		All patients (n=40)
**Median age, years (range)**		60 (33-82)
**Gender**		
Male		23 (57.5%)
Female		17 (42.5%)
**ECOG**		
0		28 (70%)
>0		12 (30%)
**Surgery**		19 (47.5%)
**anti-EGFR antibody**		16 (40%)
**anti-VEGF**		20 (50%)
**HAI Pump**		24 (60%)
**Tumor Volume cm^3^ (range)**		281 (6-1790)
**Largest lesion cm (range)**		4.9 (1.6-15)
**Number of lesions**		
≤ 3		18 (45%)
> 3		22 (55%)
**CEA (range)**		1032 (3-23938)
**Glass beads**		15 (37.5%)
**Stasis**		9 (22.5%)
**Pathway**	**Mutation**	
WT	* WT*	16 (40%)
MAPK	* KRAS*	14 (35%)
	* NRAS*	2 (5%)
	* BRAF*	2 (5%)
	* MEK1*	0 (0%)
PI3K	* PIK3CA*	9 (22.5%)
	* AKT1*	1 (2.5%)

Abbreviations: WT=wild type, ECOG=Eastern Cooperative Oncology Group Performance Status, HAI=Hepatic arterial infusion pump, CEA=carcinoembryonic antigen.

Complications were catalogued per Common Terminology Criteria for Adverse Events as follows: 7 grade 1 events, 2 grade 2 events, 2 grade 3 events, and 1 grade 4 event. The complications of grades 2-4 were managed as follows: dehydration (n=1) that was treated with intravenous hydration; pain (n=1) that was treated with intravenous pain medicine; non-target embolization to the duodenum (n=1) based on Brehmsstrahlung scan showing extrahepatic distribution that was treated with prolonged course of omeprazole and sucralfate; hyperbilirubinemia (n=1) in a patient with a biliary stent that required ERCP and stent revision; and cholangitis (n=1) that was treated with hospital admission and intravenous antibiotics. No procedure related deaths were recorded. There were no deaths recorded within 30 days.

**Figure 2:**
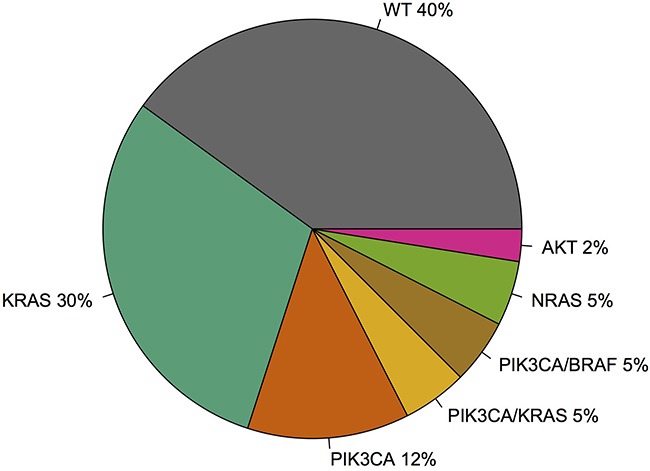
Summary of mutations identified in the cohort

**Figure 3:**
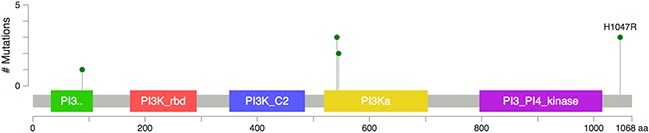
Mis-sense PIK3CA mutations identified in 9/28 patients including exon 1 (R88Q, n=1), exon 9 (E542K and E545K, n=5) and exon 20 (H1047R, n=3)

There were 18/40 (45%) patients with mutations in the MAPK pathway, including 14 patients with *KRAS* mutation, two patients with *NRAS* mutation, and two patients with *BRAF* mutation. There were 10/40 (25%) patients with mutations in the PI3K pathway including 9 patients with *PIK3CA* mutation and one patient with *AKT1* mutation. Four patients harbored concurrent PI3K and MAPK pathway mutations: two patients had both a *PIK3CA* mutation and a *KRAS* mutation and two patients had both a *PIK3CA* mutation and a *BRAF* mutation. Figure 2 summarizes the mutation data. Figure 3 summarizes the *PIK3CA* mutations identified including exon 20 (H1047R, n=3), exon 9 (E542K and E545K, n=5), and exon 1 (R88Q, n=1) [22]. There were no *MEK1* mutations identified.

**Figure 4:**
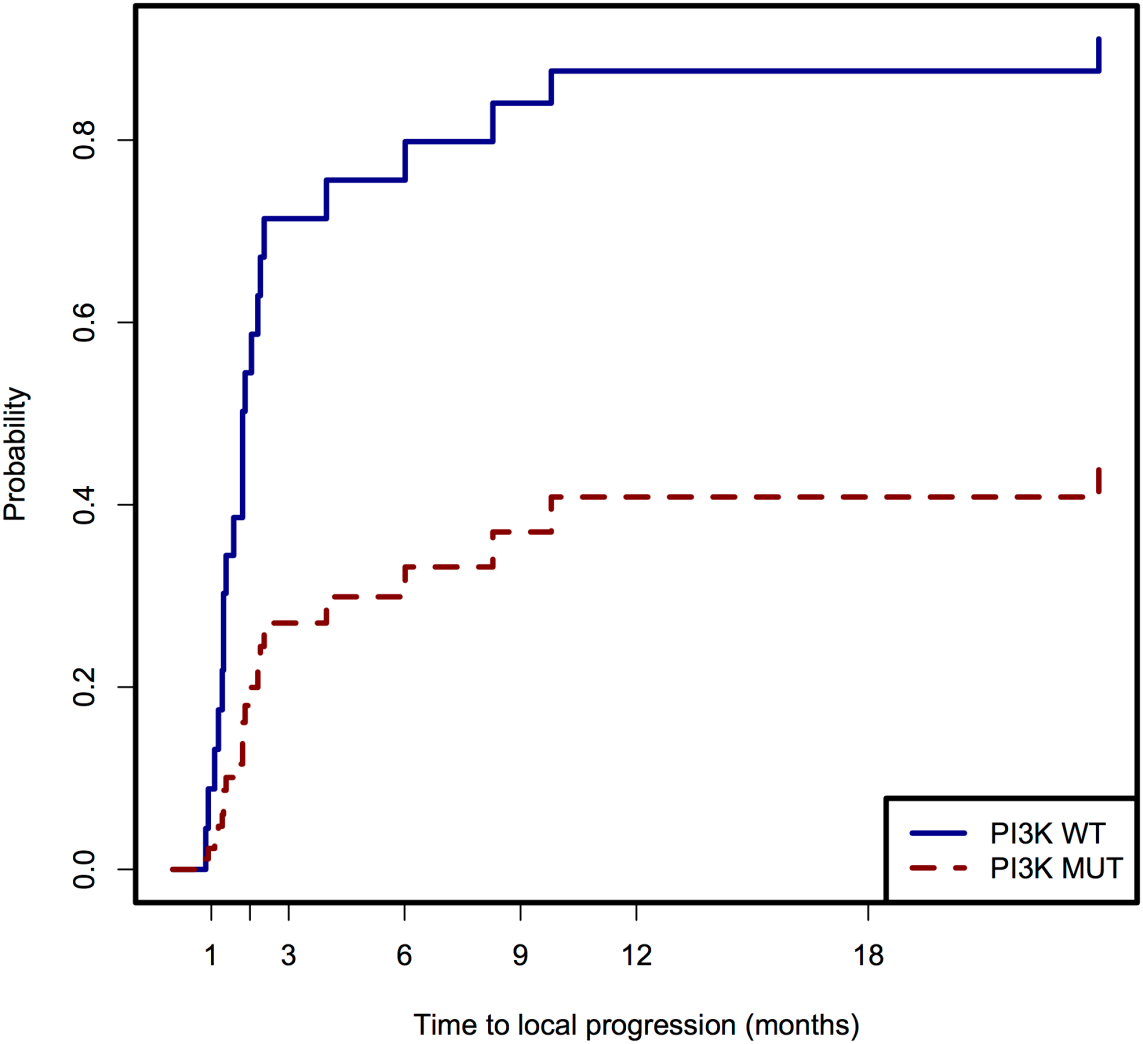
Time to local progression after RE in patients with wild type PI3K pathway and patients with mutations in the PI3K pathway

Univariate analysis of TTLP is summarized in Table 2. Presence of an activating mutation in the PI3K pathway was associated with longer TTLP (p=0.031, sHR 0.31, 95% CI: 0.11-0.90). There were no other significant variables in the univariate analysis. The cumulative incidence function generated from the competing risk univariate analysis is presented in Figure 4. The 6-month and 12-month cumulative incidence of local progression was 76% and 92% in the PI3K wild type group compared with 33% and 55% in the PI3K mutated group.

**Table 2:** Univariate analysis of time to local progression with death as competing risk

		p-value	sHR	95% CI
**Age**		0.053	0.25	0.064-1.02
**Gender**	**Male**	0.7	0.87	0.43-1.76
	**Female**			
**ECOG**	**0**	0.96	1.02	0.51-2.03
	**> 0**			
**Pump**		0.68	1.14	0.60-2.16
**Hepatic surgery**		0.2	0.65	0.33-1.26
**Anti-EGFR antibody**		0.83	0.92	0.42-2.01
**Anti-VEGF**		0.6	0.84	0.43-1.62
**CEA**		0.2	1.09	0.95-1.26
**Glass beads**		0.35	1.33	0.73-2.43
**Stasis**		0.84	0.93	0.46-1.87
**Tumor volume**		0.23	1.14	0.92-1.4
**Largest lesion**		0.35	1.33	0.73-2.42
**Number of lesions**	**≤ 3**	0.15	1.69	0.83-3.42
	**> 3**			
**MAPK pathway**		0.12	1.71	0.87-3.37
**PI3K pathway**		0.031	0.31	0.11-0.90

Abbreviations: sHR=subdistribution hazard ratio, CI=confidence interval.

Table 3 lists results from the multivariate competing risks proportional hazards model of TTLP using the 4 covariates (PI3K pathway mutation status, MAPK pathway mutation status, number of lesions, and age) included in the model based on the univariate analysis with p<0.15 (see Materials and Methods section). Mutation in PI3K pathway remained statistically significant (p=0.015, sHR=0.27, 95% CI: 0.096-0.77). Number of lesions, age, and MAPK pathway mutation status were not significant in the multivariate analysis.

**Table 3:** Multivariate analysis of time to local progression with death as competing risk using backward selection with p<0.05 as cut-off

	p-value	sHR	95% CI
**PI3K pathway**	0.015	0.27	0.096-0.77
**Number of lesions**	0.055	2.00	0.99-4.05

## DISCUSSION

The MAPK and PI3K signaling pathways are commonly mutated in patients with CLM and affect response to targeted therapies, but the effect of these mutations on response to RE remains unknown. In our series of patients, we found that the presence of a mutation in the PI3K signaling pathway was an independent predictor of longer TTLP. Patients with mutations in this pathway had significantly decreased cumulative incidence of local progression (33%) compared with patients with wild type PI3 pathway genes (76%) at 6 months. Whether these responses translate to improved patient overall survival requires further investigation.

The relationship between PI3K pathway mutations and radiation sensitivity is complex. The TME trial demonstrated that patients with non-irradiated stage 1 to 3 rectal cancer with *PIK3CA* mutation had significant increased risk of local recurrence [23] and that the relative benefit from preoperative radiation was 3 times greater among patients with *PIK3CA* mutations compared with PIK3CA wild type [24]. Yard et al recently reported that mutations that activate the PI3K/AKT pathway were associated with radiation sensitivity [25]. In other reports PI3K/AKT activation has been associated with radiation resistance [26, 27]. The variability in the literature may be related to differing cellular contexts and/or differing mutations within the *PIK3CA* gene (see Figure 3).

The mechanism by which PI3K mutations might confer radiation sensitivity is unknown. PI3K pathway mutations promote survival by increasing activity of this signaling cascade. The simplest explanation of our result is that this survival benefit is more easily inhibited by radiation compared with MAPK and other pathway mutations. Interestingly the two patients with both *PIK3CA* and *BRAF* mutations progressed locally faster (within 3 months) than patients with only *PIK3CA* mutations or combined *PIK3CA* and *KRAS* mutations, pointing to either differing radio-sensitivity of *BRAF* and *KRAS* mutations or other complex interactions via cross-talk between the MAPK and PI3K pathways [28]. PI3K pathway inhibitors are associated with radio-sensitivity [29], suggesting a potential role for adjuvant therapy at the time of RE.

Intra-tumoral heterogeneity represents a challenge for any correlative study of outcome and mutation status. Our cohort included specimens analyzed on either the primary site (n=17), a liver metastasis (n=20), a lymph node metastasis (n=2), or a lung metastasis (n=1). Analyzing tumor samples from the CAPRI-GOIM trial, Normanno et al found that *KRAS* and *NRAS* mutations were present in a majority of tumor cells, but *PIK3CA* and *BRAF* mutations were only in a fraction of tumor cells [30], suggesting that biopsies from the site of the planned treatment should be performed optimally when feasible. However, for mutations that occur early in CRC tumorigenesis, there is high concordance between primary and metastatic sites [31]. There is evidence to suggest PIK3CA mutations occur early in tumorigenesis [32, 33], possibly at the transition between adenoma and carcinoma [34]. Moreover, a direct comparison of matched primary and metastatic tumors demonstrated high genomic concordance [35].

There are several important limitations to our study. First, this is a retrospective study with a small number of patients. Our result is exploratory and should be validated in a separate cohort. The population was heterogeneous and included patients with wide range of tumor burden. Patients were identified based on availability of molecular testing at a single institution, which likely introduced a bias in our cohort. Despite these limitations, the significant findings of the current study support further prospective studies to evaluate the role of the MAPK and PI3K signaling pathways in determining response to RE.

In conclusion, we find that the presence of a mutation in the PI3K signaling pathway in CLM is an independent predictor of longer time to local progression after RE. Future prospective studies will help better delineate these potential prognostic markers in the setting of RE.

## MATERIALS AND METHODS

### Patient cohort and study design

This was a retrospective, single-center study that included consecutive patients who underwent RE for mCRC and underwent gene mutation testing. The study was approved by the institutional review board with informed consent waived and was compliant with the Health Insurance Portability and Accountability Act. We performed an institutional database search that included consecutive patients from January 1, 2010 through October 1, 2016 with CRC liver metastases treated with RE and that had had tumor specimens evaluated for mutation status of a subset of genes (see Mutation Analysis subsection).

### Covariates

Patient clinical characteristics were collected (MB) including age, gender, prior treatments (surgery, hepatic arterial intrahepatic pump, systemic chemotherapy, targeted therapy), Eastern Cooperative Oncology Group (ECOG) performance status, tumor volume, and pre-treatment carcinoembryonic (CEA) value.

### Tissue acquisition and mutational analysis

Patients with CRC have their tumor tested for downstream activators of the EGFR signaling pathway at our institution as part of standard of care. Tumor specimens were obtained via primary site specimen (17/40), liver biopsy (20/40), lymph node biopsy (2/40), or lung metastasis biopsy (1/40). After microscopic examination confirmed the diagnosis of adenocarcinoma, tissue was sent to a molecular diagnostic laboratory in the Department of Pathology for extraction of genomic DNA. All samples were determined to have adequate DNA quality prior to testing. Tumors were genotyped using (a) the Sequenom Mass Array system (Sequenom, Inc.), where samples are tested in duplicate using multiplexed assays to interrogate mutations in hotspots of *KRAS*, *BRAF*, *NRAS*, *MEK1, PIK3CA*, and *AKT1*[36] or (b) a previously reported hybridization capture-based next generation sequencing assay for targeted deep sequencing of all exons and selected introns of key cancer genes [37]. For the next generation sequencing assay that includes 8/40 (20%) of samples, we only include data related to the hotspot mutations tested in the Sequenom assay.

### Radioembolization

The decision to perform RE was reached by consensus between the interventional radiologist, medical oncologist, and surgeon, which form the core of the colorectal cancer disease management team at our institution. All patients had progressed despite previous treatments that in all cases included multiple lines of cytotoxic chemotherapy and in many cases included hepatic resection, ablation, and/or molecular therapy. RE was performed under moderate sedation using fluoroscopic guidance by a fellowship-trained interventional radiologist with at least 6 years of experience (including CS, HY, EZ). Complications were categorized using the Society of Interventional Radiology (SIR) guidelines [38]. Major complications were those that increased the level of care or required hospitalization. All other complications were considered minor. Pre-procedural baseline imaging with triphasic computed tomography (CT) in all cases was available for accurate staging of disease extent and for calculation of liver and tumor volumes. RE was performed using a microcatheter and insoluble biocompatible resin or glass spheres (Sirspheres®, Sirtex SIR-Spheres Pty Ltd, Lane Cove, Australia and Theraspheres®, MDS Nordion, Ottawa, Ontario, Canada). All patients underwent a standard pretreatment workup that comprised clinical evaluation, laboratory and imaging assessment, and a mapping procedure with technetium 99m macro-aggregated albumin. Technical success was defined as delivery of the entire prescribed dose and no stasis.

### Time to local hepatic progression

Time to local hepatic progression (TTLP) was assessed (EZ, MB, by consensus) based on Response Evaluation Criteria in Solid Tumors (RECIST 1.1) criteria at the previously treated site. The TTLP was defined as the time from initial RE to disease progression at a treated portion of the liver, and was evaluated at each follow-up CT or MRI contrast study.

### Statistical analysis

We used a standard competing risks proportional hazards model [39] to analyze TTLP with death as a competing risk and to obtain a predicted cumulative incidence function. Univariate analysis was performed using this model, and covariates with a *p≤0.15* were included in the multivariate analysis. Backward selection with a cutoff of *p=0.05* was performed to select significant predictors of outcome on multivariate analysis. Competing risk analysis was performed by R software. Tumor volume, largest lesion size, pretreatment CEA level and age were analyzed as continuous variables to avoid imposing arbitrary thresholds.
